# Spectrum of movement disorders and neurotransmitter abnormalities in paediatric *POLG* disease

**DOI:** 10.1007/s10545-018-0227-7

**Published:** 2018-08-30

**Authors:** A. Papandreou, S. Rahman, C. Fratter, J. Ng, E. Meyer, L. J. Carr, M. Champion, A. Clarke, P. Gissen, C. Hemingway, N. Hussain, S. Jayawant, M. D. King, B. J. Lynch, L. Mewasingh, J. Patel, P. Prabhakar, V. Neergheen, S. Pope, S. J. R. Heales, J. Poulton, Manju A. Kurian

**Affiliations:** 1 Molecular Neurosciences, Developmental Neurosciences Programme, UCL Great Ormond Street Institute of Child Health, 30 Guildford Street, London, WC1N 1EH UK; 2 Department of Neurology, Great Ormond Street Hospital for Children, London, UK; 3 Genetics and Genomics Medicine Programme, UCL Great Ormond Street Institute of Child Health, London, UK; 4 Mitochondrial Research Group, Genetics and Genomic Medicine Programme, UCL Great Ormond Street Institute of Child Health, London, UK; 5 Metabolic Department, Great Ormond Street Hospital for Children, London, UK; 6 Oxford Medical Genetics Laboratories, Oxford University Hospitals NHS Foundation Trust, Oxford, UK; 7 Department of Inherited Metabolic Disease, Evelina London Children’s Hospital, London, UK; 8 Paediatric Neurology Department, St George’s University Hospital, London, UK; 9 UCL-MRC Laboratory of Molecular Cell Biology, London, UK; 10 Department of Paediatric Neurology, University Hospital of Leicester, Leicester, UK; 11 Department of Paediatric Neurology, John Radcliffe Hospital, Oxford, UK; 12 Department of Paediatric Neurology and Clinical Neurophysiology, Children’s University Hospital, Temple Street, Dublin, Ireland; 13 Department of Neurology and Clinical Neurophysiology, Children’s University Hospital, Temple Street, Dublin, Ireland; 14 Department of Paediatric Neurology, Imperial College Healthcare NHS Trust, London, UK; 15 Department of Paediatric Neurology, Bristol Royal Hospital for Children, Bristol, UK; 16 Neurometabolic Unit, National Hospital for Neurology and Neurosurgery, London, UK; 17 Department of Paediatric Laboratory Medicine, Great Ormond Street Hospital for Children, London, UK; 18 Nuffield Department of Women’s and Reproductive Health, University of Oxford, The Women’s Centre, Oxford, UK

## Abstract

**Objectives:**

To describe the spectrum of movement disorders and cerebrospinal fluid (CSF) neurotransmitter profiles in paediatric patients with *POLG* disease.

**Methods:**

We identified children with genetically confirmed *POLG* disease, in whom CSF neurotransmitter analysis had been undertaken. Clinical data were collected retrospectively. CSF neurotransmitter levels were compared to both standardised age-related reference ranges and to non-*POLG* patients presenting with status epilepticus.

**Results:**

Forty-one patients with *POLG* disease were identified. Almost 50% of the patients had documented evidence of a movement disorder, including non-epileptic myoclonus, choreoathetosis and ataxia. CSF neurotransmitter analysis was undertaken in 15 cases and abnormalities were seen in the majority (87%) of cases tested. In many patients, distinctive patterns were evident, including raised neopterin, homovanillic acid and 5-hydroxyindoleacetic acid levels.

**Conclusions:**

Children with *POLG* mutations can manifest with a wide spectrum of abnormal movements, which are often prominent features of the clinical syndrome. Underlying pathophysiology is probably multifactorial, and aberrant monoamine metabolism is likely to play a role.

**Electronic supplementary material:**

The online version of this article (10.1007/s10545-018-0227-7) contains supplementary material, which is available to authorized users.

## Introduction

Mitochondrial DNA (mtDNA) depletion syndromes (MDDS) are caused by defects in mtDNA maintenance due to mutations in nuclear genes which affect either mitochondrial deoxyribonucleoside triphosphate supply or components of the mtDNA replication machinery (Rahman and Poulton 2009). DNA polymerase γ (pol γ) is essential for mtDNA replication and repair. Loss-of-function mutations of *POLG*, encoding the catalytic subunit of pol γ, result in MDDS with evidence of reduced mtDNA content or abnormal mtDNA (multiple mtDNA deletions or point mutations) in affected tissues (Cohen and Naviaux 2010).

*POLG*-related disease is clinically heterogeneous. In infancy and early childhood, Alpers syndrome (also referred to as Alpers–Huttenlocher syndrome) is the most frequent clinical presentation (Cohen and Naviaux 2010). However, there is a broad phenotypic spectrum, ranging from infantile severe encephalopathy and liver failure to later-onset external ophthalmoplegia, ataxia, myopathy and axonal sensorimotor neuropathy. Epilepsy is a major feature in most cases (Cohen and Naviaux 2010). Movement disorders are commonly described (Morten et al. 2007; Cohen and Naviaux 2010), with parkinsonism most commonly reported in adult patients (Martikainen et al. 2016). In this study, we aimed to describe the clinical spectrum of movement disorders and cerebrospinal fluid (CSF) neurotransmitter profiles in children with *POLG* mutations.

## Methods

### Patient ascertainment

Paediatric patients (16 years or younger) with confirmed biallelic *POLG* mutations were retrospectively identified from the Oxford Rare Mitochondrial Disease Service for Adults and Children database, established in 2006. All cases identified between 2006 and 2013 were included in the study. Prior to genetic confirmation, some patients had CSF neurotransmitter analysis as part of routine diagnostic investigation. These patients were identified from the UK CSF Neurotransmitter Service database. Clinical information was ascertained from (i) standardised proformas completed for diagnostic CSF and genetic testing and (ii) patient hospital records, where available (see supplementary data).

For comparative analysis, CSF neurotransmitter profiles of non-*POLG* patients admitted to a single paediatric intensive care unit (PICU) from August 1999 to November 2011 were reviewed. All patients who had neurotransmitter analysis secondary to non-*POLG*-related status epilepticus were included in the study.

### *POLG* mutational analysis

*POLG* gene sequencing was performed as previously described (Ashley et al. 2007).

### CSF metabolite analysis

CSF was collected by lumbar puncture using standardised protocols and neurotransmitters were measured by high-performance liquid chromatography, as previously described (Hyland et al. 1993; Aylett et al. 2013).

## Results

### Case ascertainment (supplementary data)

In total, 41 paediatric patients with *POLG* mutations were identified. Twenty of these patients had a documented non-epileptic movement disorder (Tables 1 and 2) and were further studied. The clinical details of eight patients have been published previously (Morten et al. 2007; McCoy et al. 2011; Allen et al. 2014; Rajakulendran et al. 2016; Hikmat et al. 2017).

**Table 1:** Clinical, radiological and genetic findings in the *POLG* mutation-positive cohort. The most common mutation encountered in *POLG* disease, p.(Ala467Thr) (Rajakulendran et al. 2016), was identified as (at least) one of the two disease-causing mutations in 14/20 patients. EPC = epilepsia partialis continua, m = months, Pt = patient, URTI = upper respiratory tract infection, y = years

Pt	Onset	Mode of presentation	Movement disorder phenotype	MRI brain	Neurotransmitters	*POLG* mutations
D1	8 m	Choreoathetosis EPC 3 months later (Morten et al. 2007)	Choreoathetosis, dystonia; continuous, generalised. Orolingual dyskinesias	Normal	Normal	c.1879C>T; p.(Arg627Trp); c.2740A>C; p.(Thr914Pro)
D2	10 m	Left focal status (Hikmat et al. 2017)	No information	Obstructive hydrocephalus (persistent Blake’s pouch cyst)	Abnormal	c.2420G>A; p.(Arg807His); c.3154G>A; p.(Gly1052Ser)
D3	10 m	Myoclonic jerks post viral illness EPC 33 days later (Allen et al. 2014)	Non-epileptic myoclonus; continuous, present in sleep	Normal	Abnormal	c.1399G>A; p.(Ala467Thr); c.2740A>C; p.(Thr914Pro)
D4	11 m	Hypotonia, mild motor delay Right focal status at 11 m	No information	Leptomeningeal enhancement	Abnormal	c.1399G>A; p.(Ala467Thr); c.2542G>A; p.(Gly848Ser)
D5	11 m	Post-infectious encephalopathy, seizures, regression (Hikmat et al. 2017)	Choreoathetosis, nystagmus, myoclonus (epileptic and non-epileptic); intermittent, not present in sleep	Dentate nuclei abnormalities, subdural effusions, dural enhancement	Abnormal	c.1399G>A; p.(Ala467Thr); c.2542G>A; p.(Gly848Ser)
D6	13 m	Hypotonia, mild motor delay Subsequent EPC at 13 m	No information	Restricted diffusion bilateral perirolandic and hippocampal regions	Abnormal	c.1399G>A; p.(Ala467Thr); c.2897T>G; p.(Leu966Arg)
D7	13 m	Status epilepticus, encephalopathy, stroke-like episodes (Hikmat et al. 2017)	Dystonia, myoclonus, chorea, tremor; intermittent, not present in sleep	Metabolic infarct of right occipital lobe	Abnormal	c.1399>A; p.(Ala467Thr); c.2740A>C; p.(Thr914Pro)
D8	13 m	Myoclonic status epilepticus	No information	No information	Abnormal	c.1399G>A; p.(Ala467Thr); c.2554C>T; p.(Arg852Cys)
D9	13 m	Status epilepticus after URTI	Chorea, myoclonus; continuous, sometimes present in sleep, worsened by illness/seizures	Grey matter abnormal signal left parietal lobe and bilateral cerebral hemispheres	Abnormal	c.2243G>C; p.(Trp748Ser); c.2740A>C; p.(Thr914Pro)
D10	13 m	EPC, movement disorder (Hikmat et al. 2017)	Choreoathetosis, myoclonus (epileptic and non-epileptic); intermittent, myoclonic jerks sometimes in sleep, worsened by illness	Volume loss; abnormal signal left insula, hippocampus, occipital cortex, thalamus	Abnormal	c.3286C>T; p.(Arg1096Cys), homozygous mutation
D11	14 m	Myoclonic status epilepticus	Myoclonus (epileptic)	Volume loss; abnormal signal right parietal cortex, insula, paracentral lobule, thalamus	Abnormal	c.1399G>A; p.(Ala467Thr); c.1283T>C; p.(Leu428Pro)
D12	18 m	Left focal status epilepticus	Choreoathetosis; continuous but improved in sleep, worsened by illness/seizures	Abnormal thalamic signal	Abnormal	c.1399G>A; p.(Ala467Thr); c.3417C>G; p.(Tyr1139*)
D13	22 m	Encephalopathy; status epilepticus	Chorea, myoclonus, restless in sleep	Abnormal thalamic signal	Abnormal	c.1399G>A; p.(Ala467Thr); c.2542G>A; p.(Gly848Ser)
D14	23 m	Hypotonia, ataxia, tremor; developed EPC at 4 years	Ataxia, tremor; intermittent, not present in sleep, no obvious triggers. After EPC: myoclonus (epileptic and non-epileptic)	Normal	Abnormal	c.1399G>A; p.(Ala467Thr); c.2403G>C; p.(Trp801Cys)
D15	17 m	Ataxia; status epilepticus later at 43 months (McCoy et al. 2011)	Truncal ataxia. After status episode: nystagmus, tremor; intermittent, not present in sleep	Normal initially. After EPC: abnormal right thalamic signal	Normal	c.1252T>C; p.(Cys418Arg); c.1399G>A; p.(Ala467Thr)
D16	10 m	Abnormal liver function, lactic acidosis, encephalopathy	Dystonia	No information	Not done	c.1399G>A; p.(Ala467Thr); c.2740A>C; p.(Thr914Pro)
D17	18 m	Focal status epilepticus, movement disorder, high CSF lactate	No specific information	No information	Not done	c.1399G>A; p.(Ala467Thr); c.2542G>A; p.(Gly848Ser)
D18	26 m	Myoclonic epilepsy, nystagmus, hypotonia, raised serum lactate; acute liver failure after sodium valproate	Ataxia	No information	Not done	c.2125C>T; p.(Arg709*); c.2243G>C; p.(Trp748Ser)
D19	6 y	Pre-existing developmental delay. Drop attacks, myoclonus and ataxia	Ataxia, myoclonus	MRI abnormal (no further information)	Not done	c.2243G>C; p.(Trp748Ser); c.2542G>A; p.(Gly848Ser)
D20	16 y	Visual disturbances, sensory ataxia and myoclonus (Rajakulendran et al. 2016; Hikmat et al. 2017)	Ataxia, myoclonus	No information	Not done	c.1399G>A; p.(Ala467Thr), homozygous

**Table 2:** CSF biochemistry of *POLG* and PICU patient cohort

Patient	Diagnosis	Age NT tested	CSF Protein (g/L)	CSF Lactate (mmol/L)	HVA (nmol/L)	5-HIAA (nmol/L)	HVA/5-HIAA	3-OMD (nmol/L)****	5-MTHF (nmol/L)	Neopterin (nmol/L)	BH4 (nmol/L)	BH2 (nmol/L)
D1	*POLG* disease (Morten et al 2007)	8m	No information	2.4 (1.8-2.9)	456 (176-851)	180 (68-451)	2.5	ND	187 (72-305)	10 (7-65)	40 (19-56)	7.8 (0.4-13.9)
D2	*POLG* disease	10m	No information	**4.17 (0.8-2.9)** ^c^	**955** (176-851)^c^	**589** (68-451)^c^	1.6	ND	142 (72-305)	**68 (7-65)**	**9 (19-56)** ^d^	**15.2** (0.4-13.9)^c^
D3	*POLG* disease (Allen et al 2014)	11m	**0.52** (0.15-0.45)^c^	Normal	651 (176-851)	287 (68-451)	2.3	134 (<300)	170 (72-305)	**94** (7-65)^c^	**65 (19-56)** ^c^	10.3 (0.4-13.9)^c^
D4	*POLG* disease	11m	No information	**High**	**1486** (176-851)^c^	**751** (68-451)^c^	2.0	38 (<300)	85 (72-305)	65 (7-65)	27 (19-56)	**16.8** (0.4-13.9)
D5	*POLG* disease	12m	**1.03** (0.15-0.45)^c^	**2.4** (0.8-1.9)^c^	**899** (154-867)^c^	**436** (89-367)^c^	2.1	ND	127 (72-305)	13 (7-65)	45 (8-57)	10.2 (0.4-13.9)
D6	*POLG* disease	13m	Normal	Normal	**1168** (154-867)^c^	**493** (89-367)^c^	2.4	32 (<50)	**56** (72-305)^d^	**85** (7-65)^c^	36 (8-57)	12.5 (0.4-13.9)
D7	*POLG* disease	13m	No information	**2.3** (0.8-1.9)^c^	765 (154-867)	330 (89-367)	2.3	32 (<50)	204 (72-305)	**81** (7-65)^c^	**59 (8-57)**	13.3 (0.4-13.9)
D8	*POLG* disease	13m	No information	No information	**938** (154-867)^c^	**429** (89-367)^c^	2.1	**85** (<50)^c^	ND	ND	ND	ND
D9	*POLG* disease	13m	No information	No information	250 (154-867)	106 (89-367)	2.4	ND	144 (72-305)	20 (7-65)	32 (8-57)	6.5 (0.4-13.9)
D10	*POLG* disease	13m	**0.81** (0.15-0.45)^c^	1.6 (0.8-1.9)	**902** (154-867)^c^	320 (89-367)	2.8	ND	76 (72-305)	46 (7-65)	21 (8-57)	9.6 (0.4-13.9)
D11	*POLG* disease	14m	No information	**High**	793 (154-867)	**440** (89-367)^c^	1.8	**129** (<50)^c^	89 (72-305)	**188** (7-65)^c^	41 (8-57)	13.6 (0.4-13.9)
D12	*POLG* disease	18m	No information	No information	757 (154-867)	306 (89-367)	2.5	ND	72 (72-305)	**196** (7-65)^c^	54 (8-57)	**14.9** (0.4-13.9)
D13	*POLG* disease	22m	No information	No information	**1733** (154-867)^c^	**762** (89-367)^c^	2.3	**204** (<50)^c^	**16** (72-305)^d^	**791** (7-65)^c^	**7 (8-57)**	**34.0** (0.4-13.9)^c^
D14	*POLG* disease	51m	No information	No information	293 (154-867)	**86** (89-367)	3.4	**116**(<50)^c^	53 (52-178)	41 (7-65)	57 (8-57)	8.1 (0.4-13.9)
D15	*POLG* disease (McCoy et al 2011)	43m	Normal	Normal	625 (154-867)	348 (89-367)	1.8	ND	123 (52-178)	32 (7-65)	42 (8-57)	**14.2** (0.4-13.9)
P1	Presumed infective encephalitis, UA	0.5m	0.55 (0.2-0.8)	1.1 (0.8-1.9)	543 (324-1098)	431 (199-608)	1.3	No information	ND	**141** (7-65)^c^	56 (27-105)	12.2 (0.4-13.9)
P2	Neonatal seizures, UA	0.5m	0.52 (0.2-0.8)	1.2 (0.8-1.9)	**239** (324-1098)^d^	213 (199-608)	1.1	No information	141 (72-305)	53 (7-65)	68 (27-105)	9.8 (0.4-13.9)
P3	Ohtahara's syndrome, UA	0.75m	**1.56** (0.2-0.8)^c^	1.1 (0.8-1.9)	549 (324-1098)	338 (199-608)	1.6	No information	106 (72-305)	**105** (7-65)^c^	20 (27-105)	10.1 (0.4-13.9)
P4	Presumed infective encephalitis, UA	1.5m	Blood stained	1.7 (0.8-1.9)	365 (324-1098)	**184** (199-608)	2.0	No information	130 (72-305)	**188** (7-65)^c^	27 (27-105)	**19.7** (0.4-13.9)^c^
P5	Status epilepticus and regression, UA	8m	0.38 (0.15-0.45)	1.3 (0.8-1.9)	383 (176-851)	171 (68-451)	2.2	No information	ND	**375** (7-65)^c^	45 (19-56)	**39.1** (0.4-13.9)^c^
P6	Recurrent status epilepticus, UA	8m	Blood stained	1.4 (0.8-1.9)	**1114** (176-851)^c^	**811** (68-451)^c^	1.4	No information	295 (72-305)	Bld	Bld	Bld
P7	Status epilepticus and dystonicus, UA	43m	0.18 (0.15-0.45)	ND	577 (154-867)	145 (89-367)	4.0	No information	ND	ND	ND	ND
P8	Neonatal sepsis*, UA	0.5m	Blood Stained	Insufficient	**3172** (324-1098)^c^	595 (199-608)	5.3	No information	**68** (72-305)	Bld	Bld	Bld
P9	Non-ketotic Hyperglycinaemia	2m	**0.46** (0.15-0.45)	1.4 (0.8-1.9)	577 (324-1098)	318 (199-608)	1.8	No information	103 (72-305)	Bld	Bld	Bld
P10	PNPO deficiency	2m	**1.44 (0.15-0.45)** ^c^	**2.6** (0.8-1.9)^c^	**151** (324-1098)^d^	**122** (199-608)^d^	1.2	No information	N	37 (7-65)	53 (27-105)	10.3 (0.4-13.9)
P11	Glutaric aciduria type 1	29m	Insufficient	**3.5** (0.8-1.9)^c^	425 (176-851)	244 (89-367)	1.7	No information	ND	40 (7-65)	11 (8-57)	0.4 (0.4-13.9)
P12	VGKC antibody mediated encephalitis	122m	0.16 (0.15-0.45)	1.1 (0.8-1.9)	**26** (71-565)^d^	78 (58-220)	0.33	No information	56 (46-160)	16 (7-65)	7 (9-39)	3.3 (0.4-13.9)
P13	PCH6, *RARS2* mutations identified	0.25m	0.93 (0.4-1.2)	1.5 (0.8-1.9)	**187** (324-1098)^d^	ND	ND	No information	131 (72-305)	22 (7-65)	56 (27-105)	8.9 (0.4-13.9)
P14	Possible mitochondrial disorder, UA**	0.25m	1.54	**2.5** (0.8-1.9)^c^	549 (324-1098)	**145** (199-608)^d^	3.8	No information	ND	**275** (7-65)^c^	81 (27-105)	**48.8** (0.4-13.9)^c^
P15	FIRES;possible mitochondrial disorder, UA***	83m	ND	**3.1** (0.8-1.9)^c^	377 (71-565)	**234** (58-220)	1.6	No information	123 (72-172)	**440** (7-65)^c^	15 (9-39)	**20.8** (0.4-13.9)^c^

Neurotransmitter levels are reported according to age-related reference ranges (Hyland et al 1993; Aylett et al 2013) (in brackets) in patients with POLG disease (D1-D15) and in patients with non-*POLG* related status epilepticus (P1-P15). No definitive diagnosis was achieved for P1-P7, P14 and P15. A mitochondrial disorder was confirmed in P13 and suspected in P14 and P15. Abnormal results are depicted in bold. ^c^values >10% above upper limit of the normal reference range. ^d^>10% below the lower limit of the normal reference range. Reference ranges for protein and lactate measurements are provided by the analysing laboratory but caution in their interpretation is warranted, as studies have indicated that higher age-specific upper limits could also be within the normal range (Leen et al 2012). Abbreviations: 3-OMD= 3-O-methyldopa, 5-HIAA= 5-hydroxyindoleacetic acid, 5-MTHF= 5-methyltetrahydrofolate, BH2= dihydrobiopterin, BH4= tetrahydrobiopterin, Bld=bloodstained, CSF= cerebrospinal fluid, FIRES= fever-induced refractory epileptic encephalopathy in school-aged children, HVA= homovanillic acid, LP= lumbar puncture, m= months of life, MRI= magnetic resonance imaging, ND= not done, Neo= neopterin, NT= neurotransmitters, OCB= Oligoclonal Bands, PCH6= pontocerebellar hypoplasia type 6, PNPO= pyridoxal 5′-phosphate oxidase, RARS2= arginyl-tRNA synthetase 2, RCE= respiratory chain enzymes, UA= undetermined aetiology, VGKC= voltage gated potassium channel. *On cardiac inotropic support (dopamine intravenous infusion) at the time of CSF sampling, **Blood lactate elevated 8.5 mmol/l, normal muscle RCE activity. ***POLG negative, liver/ muscle RCE: low complex IV activity. ^****^ Levels of 3-OMD in AADC deficiency range from 562 to 6507 nmol/l, mean 2250 nmol/L (personal communication, National Neurotransmitter Service, UK)

### Genetics

All 20 patients with a movement disorder had biallelic *POLG* mutations. Of these, 18/20 harboured homozygous/compound heterozygous missense mutations and two cases were compound heterozygotes for missense and nonsense mutations (Table 1).

### Age at clinical presentation

The age at neurological presentation ranged from 8 months to 16 years, with 17/20 patients presenting before 24 months of age (median age 13 months).

### Clinical features at presentation

Information regarding early clinical features was available for all 20 patients. Encephalopathy and/or status epilepticus was the most common mode of presentation (17/20 cases). Where CSF neurotransmitter analysis had also been performed, 11/15 patients presented either with status epilepticus or epilepsia partialis continua (EPC), preceded by an intercurrent infection in 2/15 cases. The remaining 4/15 patients (D1, D3, D14 and D15) presented initially with a movement disorder, although all eventually developed status epilepticus/EPC in the ensuing weeks or months. Data regarding administered antiepileptic drugs (AEDs) were limited or absent in most cases (Table 1).

### Movement disorder

Detailed information regarding movement disorder semiology was available for 15/20 patients. Of these, 11/15 had also undergone CSF neurotransmitter analysis, whereas 4/15 had no such available data. Non-epileptic myoclonus (12/15 cases), chorea and/or athetosis (7/15), and ataxia (5/15) were described most commonly, but tremor (3/15) and dystonia (3/15) were also reported (Table 1).

### Magnetic resonance brain imaging

Many patients had structural abnormalities on brain magnetic resonance imaging (MRI), with bilateral symmetrical thalamic changes evident in 5/14 (Table 1).

### CSF analysis

Lumbar puncture was undertaken in 15/20 cases. For most of these patients, CSF neurotransmitter analysis was performed soon (0–4 weeks) after initial neurological presentation. No patient had been administered levodopa prior to CSF sampling. Thirteen of these 15 patients had CSF neurotransmitter abnormalities (Tables 1 and 2). Raised homovanillic acid (HVA) was seen in 7/15 and abnormal 5-hydroxyindoleacetic acid (5-HIAA) in 8/15 cases (7/15 had high 5-HIAA, 1/15 low 5-HIAA). In fact, 6/15 cases had abnormalities of both HVA and 5-HIAA. Of note, none of the patients were on dopaminergic therapy (including inotropic support) at the time of CSF sampling. Pterin profiles were also frequently abnormal with high neopterin levels in 7/14 patients. 5-Methyltetrahydrofolate levels (5-MTHF), measured in 14 patients, were low in 2/14 cases. 3-O-methyldopa (3-OMD) levels were mildly elevated in 4/8 cases, but not as high as those seen in aromatic L-amino acid decarboxylase (AADC) deficiency (Table 2). Finally, CSF protein and lactate levels were also frequently elevated, where information was available (Table 2); CSF white cell counts were only available in 2/15 patients (D5 and D7) and normal for both cases (data not shown).

**Fig. 1:**
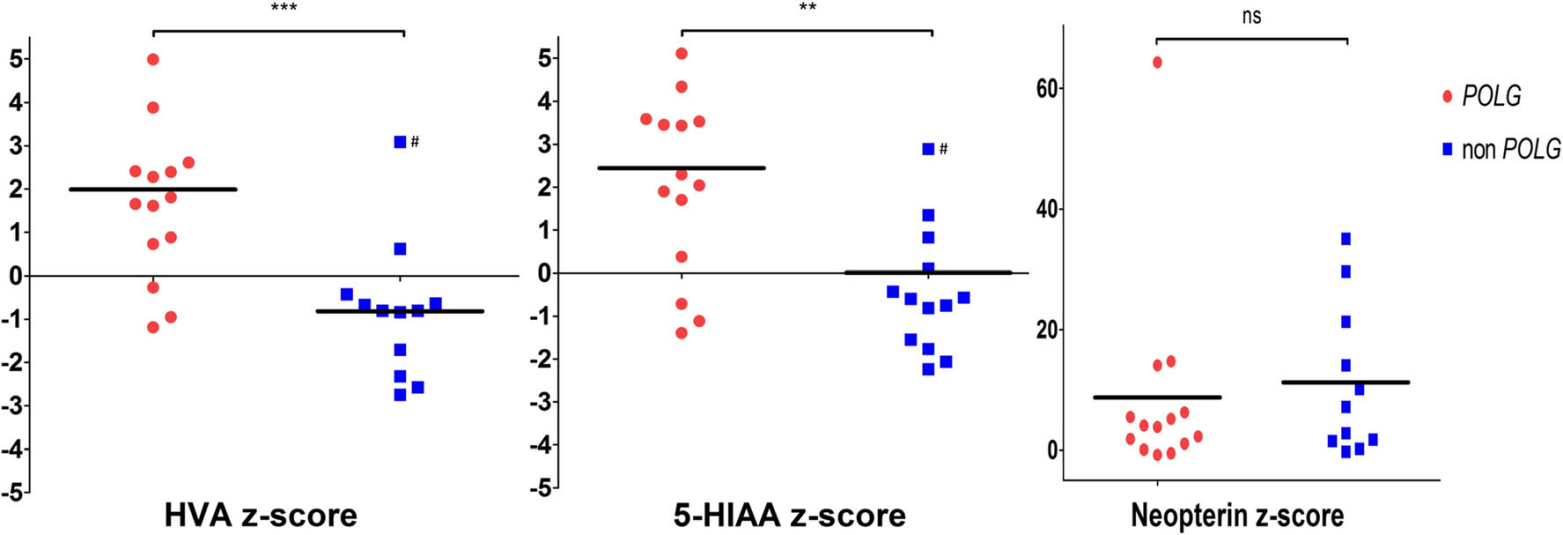
Cerebrospinal fluid (CSF) neurotransmitter abnormalities in the *POLG* and non-*POLG* cohorts. Age-specific homovanillic acid (HVA), 5-hydroxyindoleacetic acid (5-HIAA) and neopterin z-scores in patients with *POLG* disease (*red dots*) and non-*POLG*-related status epilepticus (*blue squares*) were calculated according to age-related reference ranges (Hyland et al. 1993). Patients on dopaminergic therapy at the time of CSF sample acquisition (patient P8, Table 2) were excluded from this analysis. The mean values are depicted as horizontal black lines. *POLG* HVA z-score mean = 1.99 ± 0.56, non-*POLG* HVA z-score mean = − 0.82 ± 0.46, *p* = 0.001; *POLG* 5-HIAA z-score mean = 2.45 ± 0.66, non-*POLG* 5-HIAA z-score mean = 0.01 ± 0.58, *p* = 0.01; *POLG* neopterin z-score mean = 8.71 ± 4.47, non-*POLG* neopterin z-score mean = 11.23 ± 3.75, *p* = 0.68. z-Score *p*-values were calculated using the unpaired *t*-test. *** = statistically significant (*p* = 0.001), ** = statistically significant (*p* = 0.01), ns = not statistically significant (*p* = 0.68). # = Values from patient P6, who presented with drug-resistant status epilepticus at 5 months of life. Lumbar puncture was performed at 8 months, during an intensive care unit (ICU) admission to manage seizures. *POLG* mutations and mitochondrial encephalomyopathy, lactic acidosis and stroke-like episodes (MELAS) caused by the common mitochondrial DNA (mtDNA) mutation m.3243A>G were genetically excluded

In order to determine whether the observed CSF neurotransmitter profiles in *POLG* patients were disease-specific, we undertook comparative analysis with non-*POLG* patients who had a similar disease presentation. We identified 1754 paediatric CSF neurotransmitter profiles undertaken between 1999 and 2011 in a single centre. Sixty of 1754 patients underwent CSF analysis during admission to the PICU, of which 15 were for investigation of status epilepticus (Table 2, patients P1–P15). None of these 15 cases were diagnosed with mutations in *POLG*, although *POLG* mutations were clinically suspected and subsequently excluded in P6, P7 and P15. A definitive diagnosis was achieved in 6/15 patients (P8–P13). Three of 15 patients (P13–P15) had a suspected or proven mitochondrial disorder, with CSF showing high neopterin levels in 2/3. Additionally, 3/15 patients (P1, P4 and P8) had a suspected or proven central nervous system (CNS) infection, with elevated neopterin in all three cases. Overall, CSF neopterin was elevated in 6/11 cases, where data were available. Two of 15 patients had a raised CSF HVA, one of whom was on dopaminergic therapy, whilst 4/15 had low HVA levels. 5-HIAA levels were abnormal in 5/14 cases (low in 4/14, high in 1/14). CSF 5-MTHF levels, undertaken in 9/15 patients, were low in one patient (P8) (Table 2). Age-specific (Hyland et al. 1993) CSF HVA and 5-HIAA levels were significantly higher in *POLG* patients when compared to non-*POLG* patients (*p* = 0.001 and *p* = 0.01, respectively), whereas neopterin levels were similarly elevated in both cohorts (*p* = 0.68) (Fig. 1).

## Discussion

We report the movement disorder semiology and neurotransmitter profiles in children with biallelic *POLG* mutations. *POLG* disease has previously been associated with a wide range of movement disorders. In adults and adolescents, ataxia, dystonia, chorea and myoclonus have been described but, overall, parkinsonism seems to be the most commonly encountered motor phenotype (Hinnell et al. 2012; Martikainen et al. 2016). In childhood, choreoathetosis, myoclonus and parkinsonian features have been reported (Morten et al. 2007; Cohen and Naviaux 2010). In our cohort, hyperkinetic motor phenotypes were documented in 20/41 cases, most commonly non-epileptic subcortical myoclonus and choreoathetosis. Ataxia was also frequently reported. Notably, abnormal movements sometimes preceded the onset of seizures or status epilepticus (5/20 cases), suggesting that *POLG* disease should be included in the differential diagnosis for children initially presenting with abnormal hyperkinetic movements, particularly if associated with neurodevelopmental delay, regression or epilepsy.

We observe that, where CSF neurotransmitter analysis was undertaken, the majority of *POLG* mutation-positive patients had evidence of abnormal CSF pterin and/or monoamine metabolites. Of these, many (11/15) had an initial presentation of status epilepticus and the majority (12/15) had neurotransmitter analysis performed during a period of increased seizure burden, often whilst in the PICU. Notably, children who presented with a movement disorder in the absence of seizures (patients D1, D3 and D14) had fewer neurotransmitter abnormalities than the *POLG* status epilepticus group (Table 2).

CSF HVA and/or 5-HIAA elevation was evident in 8/15 *POLG* patients. In fact, CSF monoamine levels were significantly higher in our *POLG* cohort when compared to those with non-*POLG* status epilepticus (Fig. 1, Table 2). Similar patterns of HVA and 5-HIAA elevation have been reported previously in a patient with *POLG* disease (Hasselmann et al. 2010). Importantly, normal HVA:5-HIAA ratios of 1.6–3.4 (normal range 1.0–4.0) (Ng et al. 2015) in all *POLG* patients discriminate these profiles from other primary neurotransmitter disorders, such as dopamine transporter deficiency syndrome (DTDS), where the HVA:5-HIAA ratios are commonly above 5 (Ng et al. 2015). High levels of HVA and 5-HIAA have also been reported in patients with mtDNA deletions (Pineda et al. 2006). Other mitochondrial diseases are, however, more commonly associated with low HVA and 5-HIAA levels (García-Cazorla et al. 2007; Garcia-Cazorla et al. 2008a), although not as low as in primary neurotransmitter disorders (such as tyrosine hydroxylase or aromatic L-amino acid decarboxylase deficiency), where much lower CSF levels are usually reported (Ng et al. 2015).

Overall, 7/12 *POLG* patients presenting acutely with seizures or intercurrent infections had high neopterin levels, with levels up to 12 times above the upper limit of the normal reference range (Hyland et al. 1993). Similar neopterin elevation was seen in 6/11 cases of the non-*POLG* status epilepticus cohort. BH2 and BH4 were also frequently raised in both cohorts, often in tandem with high neopterin levels. High neopterin levels are considered a biochemical marker of inflammation within the CNS and frequently encountered in conditions associated with an exaggerated or aberrant immune response, such as CNS infections, multiple sclerosis and Aicardi–Goutières syndrome (Dale et al. 2009). In keeping with CSF inflammation, CSF protein and/or lactate levels were also high in 9/15 cases, as per previous reports (Cohen and Naviaux 2010). Similar high neopterin levels have previously been reported in a case of *POLG* disease (Hasselmann et al. 2010). The underlying basis of raised pterin levels in *POLG* patients is currently unclear, but it may be related to an immune-mediated response associated with intercurrent infection, frequent seizures at the time of CSF sampling or the underlying disease itself.

Two of 14 patients had low CSF 5-MTHF levels, being moderately reduced in one patient (D6) and more markedly reduced in another (D13). Cerebral folate deficiency is reported in several types of mitochondrial disease (Pineda et al. 2006; Garcia-Cazorla et al. 2008b), including *POLG* mutations (Hasselmann et al. 2010; Rajakulendran et al. 2016), ranging from mild deficiency to more severe forms that can mimic primary folate disorders, such as those due *FOLR1* mutations (Cario et al. 2009). The mechanisms underpinning cerebral folate deficiency might include choroid plexus dysfunction, inefficient ATP-dependent transport of folate from blood into the CSF, oxidative stress (Aylett et al. 2013; Rahman 2015) or the presence of blocking-type folate receptor autoantibodies (Hasselmann et al. 2010). Folinic acid treatment sometimes leads to clinical and radiological improvement (Pineda et al. 2006), suggesting a putative link between low CSF 5-MTHF levels and observed phenotypes in these patients (Rahman 2015).

Overall, there seems to be no CSF biomarker that is universally abnormal in *POLG* patients, at least at disease onset, when CSF is most likely to be obtained; even CSF protein and lactate levels were normal in a few cases (Table 2). However, our results suggest that CSF neurotransmitter analysis might be a helpful tool to herald the possibility of *POLG* disease in affected patients.

Our study has a number of limitations. Given the retrospective nature of our work, patients were identified as having *POLG* mutations as part of clinical care and not in the context of a genetic epidemiology study, which may lead to selection bias. However, case identification took place in a nationally commissioned centre performing *POLG* diagnostic testing; hence, our results are likely to be representative of the paediatric *POLG* mutation-positive population. Additionally, there was no standardised approach to motor phenotype characterisation while, in some cases, there was insufficient data regarding concurrent AEDs administered, CSF biochemistry, movement disorder semiology and distribution. Furthermore, it is unclear whether the absence of movement disorders in 21/41 patients is a true representation or due to under-recognition and/or under-reporting. Regarding CSF biomarkers, we have not examined the neurotransmitter profiles in *POLG* patients who do not manifest abnormal involuntary movements, and, thus, more studies in this area are warranted. Finally, it is conceivable that whole genome sequencing analysis could help to elucidate the role of additional genetic factors contributing to phenotypic variability in our patient cohort. Overall, despite the above caveats, our findings certainly highlight that *POLG* disease can be associated with both movement disorders and aberrant CSF neurotransmitter profiles.

The pathophysiology of movement disorders in *POLG* disease is likely multifactorial. Firstly, previous studies have shown progressive striatonigral degeneration in *POLG* patients, especially with increasing age (Tzoulis et al. 2016). The early stages of this neurodegenerative process may lead to the abnormal motor phenotypes seen in our cohort. Additionally, the energy-depleted state of *POLG* disease could render the brain susceptible to acute focal injury triggered by epileptic seizures. The high neopterin levels documented in both *POLG* patients and controls suggest an acute process common to both groups that may potentially be linked to seizures. However, the high HVA and HIAA levels indicate specific involvement of dopaminergic and serotoninergic systems in the *POLG* patients but not the controls, and this may underpin the movement abnormalities. Further studies are now warranted in order to investigate whether these high levels are attributed to either increased production of serotonin and dopamine or accelerated monoamine degradation. The raised 3-OMD levels seen in some patients may be indicative of increased L-dopa synthesis. It is also clear that substantia nigra dopaminergic neurons are more vulnerable to defects of mtDNA maintenance than other mtDNA abnormalities (Tzoulis et al. 2016). Therefore, processes other than simple energy depletion or complex 1 deficiency probably underlie their susceptibility. For instance, substantia nigra dopaminergic neurons are specifically vulnerable to defects in mitophagy (a type of mitochondrial quality control) (Narendra et al. 2010), with genetic defects in *POLG* and Parkin, a key mitophagy protein, exerting synergistic effects in these cells (Pickrell et al. 2015).

In conclusion, hyperkinetic movement disorders are frequently encountered in children with *POLG* mutations, and may even be the presenting neurological feature, preceding the onset of seizures. Analysis of further cases may allow us to determine the diagnostic utility and biological relevance of observed CSF profiles (raised neopterin/HVA/5-HIAA/3-OMD) in a larger cohort of *POLG* patients. The mechanisms underpinning movement disorders in *POLG* disease are not fully understood; however, our report indicates that aberrant dopamine and serotonin metabolism may play a role.

## Electronic supplementary material

Below is the link to the electronic supplementary material.

ESM 1 (DOCX 399 kb)
