# Supplementary material for: Spectrum of movement disorders and neurotransmitter abnormalities in paediatric POLG disease
Source: J Inherit Metab Dis. 2018 Aug 30;41(6):1275–83. doi: 10.1007/s10545-018-0227-7 (PMC6326959; doi:10.1007/s10545-018-0227-7)
Supplement: Supplementary file 1 — (DOCX 399 kb) [file 10545_2018_227_MOESM1_ESM.docx]

**Spectrum of Movement Disorders and Neurotransmitter Abnormalities in Paediatric *POLG* disease**

**Supplementary Figure 1**

**
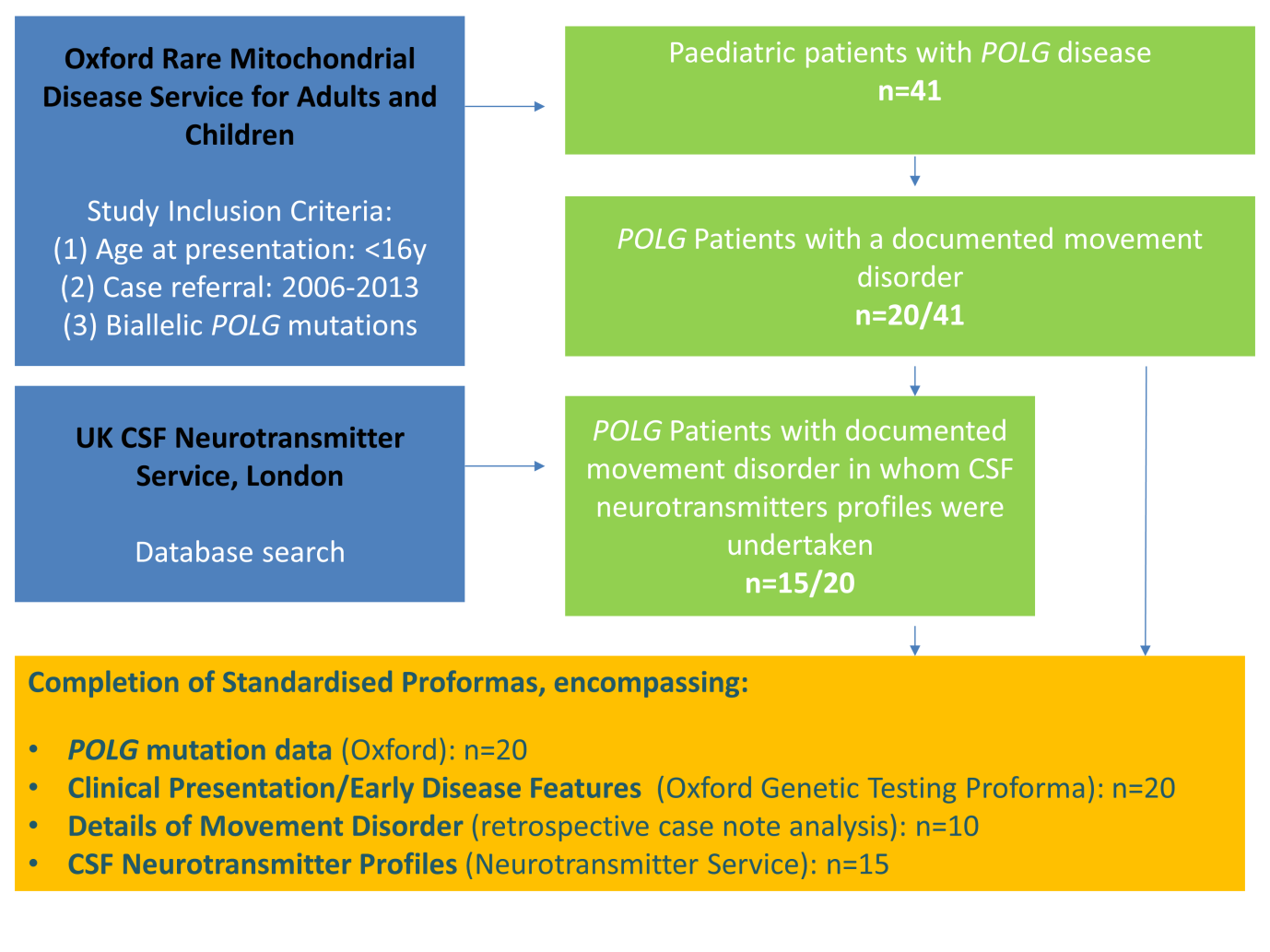
**

**Supplementary Figure 1: Case Ascertainment.** Paediatric patients with bi-allelic, disease-causing, *POLG* mutations were retrospectively identified from the Oxford Rare Mitochondrial Disease Service for Adults and Children database. Patients who had also undergone CSF neurotransmitter analysis were identified through the UK CSF Neurotransmitter Service. Patient hospital records and proformas completed prior to the diagnostic CSF and genetic testing were subsequently analysed for the evaluation of the *POLG*-related movement disorder semiology and CSF biochemistry.
